# Does digital technology reduce health disparity? Investigating difference of depression stemming from socioeconomic status among Chinese older adults

**DOI:** 10.1186/s12877-021-02175-0

**Published:** 2021-04-21

**Authors:** Aruhan Mu, Zhaohua Deng, Xiang Wu, Liqin Zhou

**Affiliations:** School of Medicine and Health Management, Tongji Medical College, Huazhong University of Science and Technology, Wuhan, Hubei, 430030 China

**Keywords:** Diversity in aging, Mental health, Digital technology

## Abstract

**Background:**

Prior studies on health disparity have shown that socioeconomic status is critical to inequality of health outcomes such as depression. However, two questions await further investigation: whether disparity in depression correlated with socioeconomic status will become larger when depression becomes severer, and whether digital technology will reduce the disparity in depression correlated with socioeconomic status. Our study aims to answer the above two questions.

**Methods:**

By using the dataset from China Health and Retirement Longitudinal Study 2015, we use quantile regression models to examine the association between socioeconomic status and depression across different quantiles, and test the moderating effect of digital technology.

**Results:**

Our study obtains four key findings. First, the negative effects of socioeconomic status on depression present an increasing trend at high quantiles. Second, Internet usage exacerbates the disparity in depression associated with education level on average, but reduces this disparity associated with education level at high quantiles. Third, Internet usage reduces the disparity in depression associated with income on average and at high quantiles. Fourth, mobile phone ownership has almost no moderating effect on the relationship between socioeconomic status and depression.

**Conclusions:**

Our findings suggest the potential use of digital technology in reducing disparity in depression correlated with socioeconomic status among middle-aged and aged individuals in developing countries.

**Supplementary Information:**

The online version contains supplementary material available at 10.1186/s12877-021-02175-0.

## Introduction

Depressive symptoms are characterized by persistent sadness and a loss of interest in all or almost all activities, accompanied by the syndromes, such as weight loss or gain, which last at least 2 weeks [1]. Depressive disorder, which has been suffered by 300 million people worldwide in 2015, is one of the main leading causes of further increase in the number of all-age years lived with disability (YLDs) in 1990 up to 2017 [2]. Depression is particularly acute in the elderly and in low- and middle-income countries (LMICs) [3] With the high prevalence of depression in the elderly and the continuous aging of people aged 45–55 years, the health system of all countries faces major challenges to ensure the well-being of aging individuals [4], especially in LMICs. In China, the depression prevalence rates of middle- and old-aged individuals are quite high, with the overall prevalence of depression ranging from 11 to 57% among people aged over 60 years [5]. Previous studies have concluded that socioeconomic status (SES) is a strong predictor of depression [5–11]. Studies in the Chinese context have identified several factors strongly associated with later-life depressive symptoms, such as education, income [5, 8], hukou [9, 12, 13] and childhood conditions [8, 11]. However, these findings are difficult to guide us in direct health interventions because we can hardly change SES. In other words, disparity in depression correlated with SES is deep-rooted and will persist. In this case, a key question is: can we weaken this deep-rooted disparity in depression through feasible means?

Digital technology, which is characterized as low cost and easy accessibility, has considerable potential to deliver public health intervention, especially in LMICs [14–16]. Thus far, positive outcomes have been reported in randomized controlled trials of digital interventions across a wide range of chronic disease outcomes, such as cell phone voice and text message interventions positively impacted on chronic disease outcomes, improving attendance rates and health-related quality of life, and was cost-effective [15]. Web-based interventions, videoconferencing, and online support groups to deliver psychotherapy have been well validated and showed efficacy on depression [17, 18]. Though prior studies [19–23] have examined the direct effect of digital technology on depression, few of them considered the moderating effect of digital technology. Instead, this paper focuses on the moderating effect and investigates whether digital technology can weaken the deep-rooted disparity in depression correlated with SES.

This study uses a quantile regression approach to provide a holistic view of how SES influences depression and how digital technology moderates the relationship between SES and depression. Our methodology has two advantages. First, quantile regression outperforms OLS for skewed distributed dependent variable. Our data are from China Health and Retirement Longitudinal Study 2015, in which the distribution of depression is right skewed. Thus, conditional mean cannot well describe the relationship between SES and depression, which makes ordinary least squares (OLS) estimates unsatisfactory. Second, quantile regression has been widely used to examine health disparity since it can provide a holistic view of the relationship between two variables [24, 25]. Quantile regression model provides us the capability to “think beyond the mean”. From a practical point of view, we are particularly concerned about the situation of severe depression. Consequently, we adopt the quantile regression approach and answer two research questions: [1] Will the disparity in depression correlated with socioeconomic status expand under severe depression cases? (2) Will digital technologies reduce the disparity in depression correlated with socioeconomic status at different quantiles?

### Theoretical background and hypotheses

Socioeconomic status is essential social origin of disparity in depression [26, 27]. The biomedical literature has generally treated SES as a unitary construct [28]. Likewise, the literature that explores the mechanisms linking life-course SES and health in later life has tended to treat SES as a latent variable [6, 11, 29]. To advance our understanding of the relationship between SES and depressive symptoms among older adults, and ultimately to foster appropriate policies and practices to improve population health, a more nuanced approach is required [28].

Income, education, and occupation are important factors of SES [5, 7, 12, 28, 30]. Early studies in developed countries have shown that education and income can predict disease onset and progression, respectively [28]. A recent analysis among European older adults shows that education and income are the SES indicators more frequently significantly associated with depression but not occupation [7]. This effect might be more significant in developing countries [5, 8, 9, 11, 30]. In China, education and income are robustly associated with later life depression [5, 8]. Also, factors such as general health during childhood and parental education are highly associated with later depression [8, 10, 11]. Good self-rated health status during childhood [8, 11] and a high level of parental education [10, 11] are negatively associated with depressive symptoms. In the Chinese context, hukou (household registration) status, which is categorized into agricultural or non-agricultural, is related to the availability of a wide range of social benefits [31]. Individuals with agricultural hukou are more likely to be farmers with a lower level of education and income than non-agricultural hukou [12]. Studies have reported that rural older adults in China have higher levels of depression than their urban peers [9, 13]. Thus, we propose that Hukou might be a key factor besides education, income, and childhood conditions.

Basing on these arguments, we propose that individual SES, which indicated by parental education, self-rated health status during childhood, education, income, and hukou, is negatively associated with depressive symptoms in later life. In this study, depression status is measured by the Center for Epidemiological Studies-Depression Scale (CES-D). The higher the score is, the severer the depression. Thus, our hypothesis is:

H1: Individual SES is negatively associated with depression in later life.

We focus on subgroups with severe depressive status, which have higher CES-D score, by estimation at high quantiles to examine association with individual SES. We propose that the disparity in depression may be larger among subgroups of older adults with severe depression status. Thus, we hypothesize the following:

H2: Disparity in depression correlated with individual SES is larger among higher quantile-level subgroups.

In the context of “formed” individual SES, human agency and resource mobilization may reshape the outcomes [27]. The growing Evidences suggest that using digital technology can help maintain social contact, enhance social support, reduce social isolation, loneliness, and depressive symptoms among older adults [23, 32–38]. Cotten et al. [32], using a large longitudinal sample from Health and Retirement Survey, find that Internet use reduced the probability of a depression state by 33%. A randomized controlled trial and quasi-experimental research of Internet training and access for older adults find a significant reduction in loneliness and depressive symptoms among participants in the intervention group [35, 37]. According to the review of qualitative evidence, digital technologies have a beneficial impact on mental health among older adults by enhancing interpersonal interaction, increasing access to resources, and empowering social inclusion [39].

Thereby, we argue that in the context of SES leading to health disparity, digital technology usage will alleviate the inequality of depression correlated with SES. We only consider individual SES factors that have been formed and are interferable at this stage, such as education, income, and hukou. In other words, digital technology usage negatively moderates the relationship of “formed” and “interferable” individual SES, including education, income, and hukou, to depression. Thus, we hypothesize the following:

H3: Digital technology usage negatively moderates the relationship of individual SES and depression.

Currently, vulnerable subgroups among the elderly have come into the sight of researchers and policymakers. Given that disadvantaged older adults may face more mobility and activity limitations, worse health status or frailty, the relative importance of digital technology for maintaining social connectedness, obtaining social support and resources may be greater [33, 34, 36, 38]. Ruppel et al. [34], using data from Wisconsin Longitudinal Study, find that e-mail usage might help older adults mitigate hearing impairments and associated depressive symptoms. Fang et al. [33], conducting telephone interviews in Hong Kong older adults, find that Internet use enhances psychological well-being among oldest-older adults, and these benefits might be particularly salient for those who were frail. Yuan [38], using data from the Shanghai Urban Neighborhood Survey find that Internet use may reduce more mental health problems in the unhealthy group. Thereby, we propose that the negatively moderate effect of digital technology usage may be strengthened among subgroups with severe depression status of older adults. Thus, we hypothesize the following:

H4: The moderating effect of digital technology usage is strengthened among higher quantile-level subgroups.

## Methodology

### Data description

The China Health and Retirement Longitudinal Study (CHARLS) is a nationally representative longitudinal survey of persons aged 45 years or older in China. The survey is conducted by the National School of Development of Peking University. The baseline wave of CHARLS conducted in 2011 covered about 10,000 households and 17,500 individuals in 150 counties and 450 villages. CHARLS respondents are followed up every 2 years by face-to-face computer-assisted personal interview [40].. Our data are obtained from the harmonized CHARLS and 2015 follow-up. Our complete dataset, in which all measured variables are not missing, contains 8853 participants.

### Measures

The primary independent variable of interest is socioeconomic status of respondents and usage of digital technology. Parental education and self-rated health status during childhood before age 16 (SRH-16) to determine the effect of childhood conditions. In this study, educational level is categorized into two groups: coded 0 for illiteracy indicating no formal education and no ability to read and write, and coded 1 for literacy, which consistent with any of the following: less than lower secondary, upper secondary, or tertiary. SRH-16 is a subjective measure of one’s health status before 16 years old and is reported on a five-point scale, ranging from 1 to 5 as follows: poor, fair, good, very good, and excellent. Income includes individual wages and bonus income from work, individual’s after tax net income earned from self-employed activity, pension income, and other income from child support or alimony or fringe benefits provided by the work place. Hukou is categorized into two groups: coded 0 for agricultural hukou (rural residence) and 1 nonagricultural hukou (urban residence). For variables with hyperdispersion property such as income, we take logarithm transformation. Besides, it is worth noting that the proportion of mothers educated is too low (less than 15%) and fathers educated is 44.1%, so we included father’s education in the main model. The results of main model included mother’s education see Additional file - Table 1.

Access to digital technology is measured by Internet usage and mobile phone ownership. Our research design considers the possible reverse causality. Independent variables, including Internet usage and phone ownership, and dependent variables from our dataset have a natural chronological order. To build our Internet usage variable, we focus on responses in the survey (1 yes/0 no) about whether the respondents have accessed the Internet in the past month. To build our mobile phone usage variable, we focus on responses in the survey (1 yes/0 no) about whether the respondents own a mobile phone.

Depression status is a dependent variable that is measured by a 10-item CES-D. This measure is used in elderly population [41]; Sun, Guo, Liu, & Gao, [54]. Eight items measure symptoms of depression frequency, and two items measure the positive affect on a four-point scale, ranging from 0 to 3. The score is assigned by totaling all item scores after reversing two items of positive affect to fit the measurement scale model, ranging from 0 to 30. From the sociological perspective of depression and considering the cultural bias in responses to the items in CES-D, the outcome is more suitable to be conceptualized as a continuum consisting of flourishing and languishing than be identified as a certain cutoff point to derive health versus illness from the physical illness model [42, 43]. Thus, in this study, CES-D score is considered as a continuous variable. And the higher the score is, the severer the depression.

Our study also includes the following individual demographics as controls: age is a continuous variable, ranging 45 years or older; gender is measured as a dichotomous variable, in which 1 equals male; marital status is a dichotomous measure, coded 1 for married and 0 for others.

### Econometric model

Our study consists of two parts: examining the association between individual SES on depression. Model 1 is specified as

$$ Doutcome={\beta}_0+{\beta}_1{edu}_{father}+{\beta}_2{srh}_{childhood}+{\beta}_3 edu+{\beta}_4 income+{\beta}_5 hukou+{\beta}_{6-8} control\ variables+e $$

where *β*_1_, *β*_2_, *β*_3_, *β*_4_, and *β*_5_ determine the associated effects of childhood conditions (such as father’s education and SRH-16) and “formed” and “interferable” SES at this stage, including education, income, and hukou.

We use model 2 to investigate the moderating effect of digital technology usage on the association between depression and “interferable” SES, aiming to reveal the intervening potential of digital technology on relation between depression and education, income, and hukou in practice.

$$ Doutcome={\beta_0}^{\prime }+{\beta_1}^{\prime }{edu}_{father}+{\beta_2}^{\prime }{srh}_{childhood}+{\beta_3}^{\prime } edu+{\beta_4}^{\prime } income+{\beta_5}^{\prime } hukou+{\beta_{6-8}}^{\prime } control\ variables+{\beta}_{DT}{usage}_{DT}+{\beta}_{DT\ast EDU} edu\times {usage}_{DT}+{\beta}_{DT\ast INCOME} income\times {usage}_{DT}+{\beta}_{DT\ast HUKOU} hukou\times {usage}_{DT}+{e}^{\prime } $$

*usage*_*DT*_ include Internet usage or mobile phone usage. *β*_*DT*_ indicate the direct effect of Internet or mobile phone usage on depressive symptoms under control of “interferable” SES and childhood conditions. *β*_*DT* ∗ *EDU*_, *β*_*DT* ∗ *INCOME*_ *and β*_*DT* ∗ *HUKOU*_ determine the moderating effects of Internet or mobile phone usage on the relationship between depression and “interferable” SES, respectively.

### Statistical analysis: quantile regression

Ordinary least-squares (OLS) estimation of the mean regression models determine how the conditional mean of Y (CES-D scores) depend on covariate X (independent variables include individual SES and digital technology usage). Quantile regression, which is not influenced by outliers, can analyze the effect of X across the various distributions of Y and provide a holistic view and robust results by calculating coefficient estimates across the various quantiles of the conditional distribution [44]. These characteristics help us to reveal the relationship between depression, SES, and digital technology in the subgroup of severe depressive symptoms. The quantile regression model is specified as

$$ {Q}_{Y_i}\left(\tau |{x}_i\right)=\alpha \left(\tau \right)+\beta \left(\tau \right){x}_i+{\beta}^{\prime}\left(\tau \right){x}_i\cdotp {z}_i+{Q}_{\tau }(u). $$

where *Y*_*i*_ are the CES-D scores of the participants, τ is a specific set of quantile level, *x*_*i*_ is the set of participants’ individual SES variables, and *z*_*i*_ is the set of participants’ digital technology usage variables. Parameter *β*(*τ*) models the direct effect of individual SES on depression, and *β*^′^(*τ*) models the moderating effect of digital technology usage. *u* represents the random error term. The quantile regression model is estimated using weighted least absolute deviation (WLAD) and performed using R package “quantreg.”

## Results

### Descriptive statistics

**Fig. 1:**
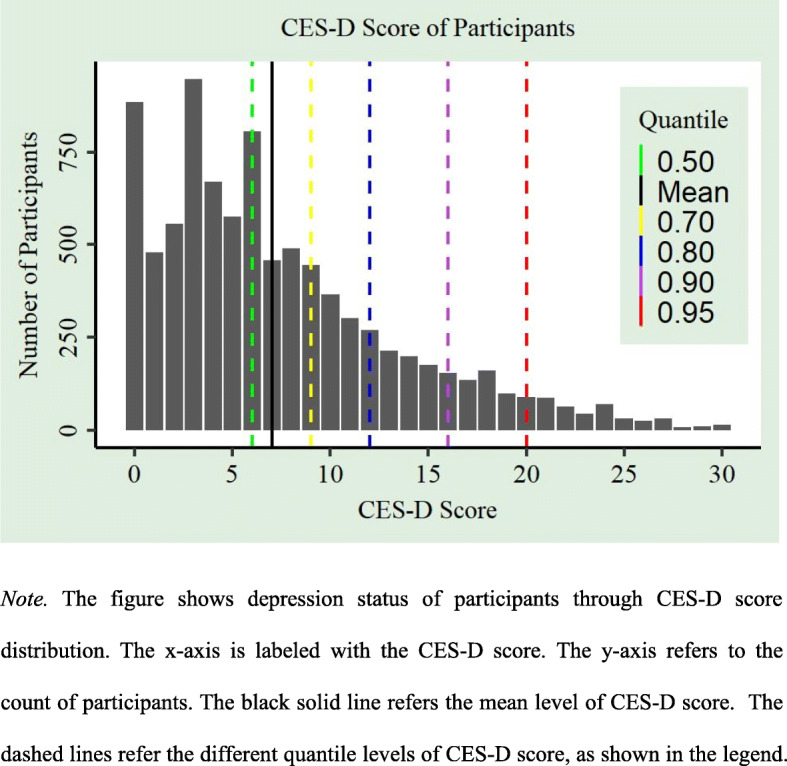
CES-D score of participants

The descriptive statistics of all variables is provided in Table 1. Figure 1 shows the CES-D scores of participants. It displays the depression status (or healthy status) of participants through the CES-D score distribution ranging 0–30 and clarifies the CES-D scores corresponding to different quantile levels.

**Table 1:** Descriptive statistics (sample *n* = 8853)

Variables	Frequency	Percent	Mean	SD
**Demographics**				
Age			60.36	10.27
Gender				
*Male*	4918	55.5%		
*Female*	3935	44.5%		
Marital				
*Married*	7217	81.5%		
*Other*	1636	18.5%		
**Individual Socioeconomic status**				
Father’s education				
*Literacy*	3907	44.1%		
*Illiteracy*	4946	55.9%		
SRH-16				
*Poor*	535	6.0%		
*Fair*	1966	22.2%		
*Good*	1691	19.1%	3.31	1.13
*Very good*	3546	40.1%		
*Excellent*	1115	12.6%		
Education				
*Literacy*	7030	79.4%		
*Illiteracy*	1823	20.6%		
Income				
*Log (income)*			8.63	1.74
Hukou				
*Agricultural hukou*	6604	74.6%		
*Non-agricultural hukou*	2249	25.4%		
**Digital technology usage**				
Internet usage				
*Yes*	880	9.9%		
*No*	7973	90.1%		
Mobile Phone usage				
*Yes*	4675	52.8%		
*No*	4178	47.2%		
**Depression status**				
CES-D score			7.31	6.10

### Socioeconomic status and depression

To test the hypotheses of the proposed model, we consider three models: (1) one baseline model, where we evaluate H1 and H2; and (2) two interaction models, where we included the interaction term of individual SES and digital technology usage to evaluate H3 and H4. Specifically, we built two interaction models to test the moderating effect of Internet usage and mobile phone usage on the relationship between SES and depression, respectively. We report the results of OLS and median regression (0.5 quantile level) to explain the average effect. Further, we report the relation between depression, SES, and digital technology usage at high quantiles (0.6, 0.7, 0.8, 0.9 quantile level) focusing on subgroups with severe depression status.

Table 2 shows the estimates of model 1 (a baseline model). SRH-16 (*β*_2_ =  − 0.444, *p* < 0.01), education (*β*_3_ =  − 0.739, *p* < 0.01), income (β_4_ =  − 0.665, *p* < 0.01), and hukou (*β*_5_ =  − 0.348, *p* < 0.05) predicted a decline in depressive symptoms. In median regression model (Q = 0.5), the above variables significantly associated with decline in depressive symptoms, similarly. **H1 is partly supported**.

**Table 2:** OLS analysis and quantile regression estimation for model 1

Variables	Dependent variable: depression (sample *n* = 8853)					
	OLS	Quantile regression				
		0.5	0.6	0.7	0.8	0.9
	(1)	(2)	(3)	(4)	(5)	(6)
**Individual socioeconomic status**						
Father’s education	−0.197	−0.181	−0.344**	−0.175	−0.093	− 0.098
	(0.132)	(0.145)	(0.170)	(0.194)	(0.243)	(0.294)
SHR-16	−0.444***	− 0.460***	− 0.486***	−0.521***	− 0.649***	− 0.676***
	(0.055)	(0.061)	(0.073)	(0.086)	(0.102)	(0.121)
Education	−0.739***	−0.730***	− 0.873***	−1.080***	−1.670***	−0.776*
	(0.174)	(0.250)	(0.253)	(0.373)	(0.320)	(0.413)
Income	−0.665***	−0.628***	− 0.753***	−0.865***	− 1.100***	−1.280***
	(0.046)	(0.055)	(0.060)	(0.075)	(0.090)	(0.106)
Hukou	−0.348**	− 0.310*	− 0.347*	− 0.505**	−0.283	− 0.365
	(0.164)	(0.164)	(0.198)	(0.220)	(0.293)	(0.344)
**Other**						
Age	0.018**	0.011	0.015	0.013	0.017	0.041**
	(0.007)	(0.008)	(0.009)	(0.011)	(0.013)	(0.016)
Gender	−1.330***	−1.290***	−1.400***	− 1.980***	−2.230***	−2.660***
	(0.133)	(0.157)	(0.181)	(0.222)	(0.249)	(0.299)
Marital	−1.400***	−1.380***	− 1.490***	−1.920***	− 2.000***	− 2.680***
	(0.161)	(0.218)	(0.235)	(0.308)	(0.294)	(0.409)
Constant	16.100***	15.000***	17.800***	21.600***	26.700***	30.700***
	(0.744)	(0.874)	(0.969)	(1.210)	(1.410)	(1.680)
Observations	8853	8853	8853	8853	8853	8853
*R*^2^	0.110					
Pseudo *R*^2^		0.595	0.596	0.595	0.606	0.601

^a^ standardize coefficients are reported; standard errors in parentheses

^b^ ***p < 0.01, **p < 0.05, *p < 0.1

At high quantiles, we find that the coefficient of father’s education is negative and significant (*β*_1_ =  − 0.344, *p* < 0.05, Q = 0.6). The effect of health status during childhood, education, and income showed significantly growing trend at high quantiles. The negative effect of hukou is increased to the highest for 0.7 quantile level (*β*_5_ =  − 0.505, *p* < 0.05, Q = 0.7). **H2 is partly supported**. The quantile regression plot of model 1 see Additional file - Figure 1.

### Moderating effect of digital technology

Table 3 shows the estimation of model 2, which evaluated the interaction effect of SES and Internet usage. First, Internet usage significantly associated with a decline in depressive symptoms (*β*_*DT*_ =  − 5.800, *p* < 0.05, OLS and *β*_*DT*_ =  − 3.500, *p* < 0.1, Q = 0.5). In the median regression model, Internet usage has positive moderating effect on relationship between education and depression (*β*_*DT* ∗ *EDU*_ =  − 1.460, *p* < 0.01, Q = 0.5). The interaction effect of income and Internet usage is significantly positive (*β*_*DT* ∗ *INCOME*_ = 0.502, *p* < 0.05, OLS and *β*_*DT* ∗ *INCOME*_ = 0.472, *p* < 0.05, Q = 0.5). As such, Internet usage will negatively moderate the relationship between income and depression. For hukou status (*β*_5_^′^), the direct effect is not significant after including digital technology usage in model. In the scenario of Internet usage, **H3 is partly supported**.

**Table 3:** OLS analysis and quantile regression estimation for model 2 (Internet usage)

Variables	Dependent variable: depression (sample *n* = 8853)					
	OLS	Quantile regression				
		0.5	0.6	0.7	0.8	0.9
	(1)	(2)	(3)	(4)	(5)	(6)
**Individual socioeconomic status**						
Father’s education	−0.168	−0.163	−0.264	−0.138	−0.118	−0.143
	(0.132)	(0.149)	(0.163)	(0.188)	(0.238)	(0.310)
SRH-16	−0.437***	−0.443***	− 0.451***	−0.526***	− 0.640***	−0.666***
	(0.055)	(0.062)	(0.070)	(0.083)	(0.099)	(0.128)
Education	−0.734***	−0.722***	− 0.874***	−1.120***	−1.660***	−0.803*
	(0.175)	(0.238)	(0.255)	(0.376)	(0.311)	(0.421)
Income	−0.685***	−0.642***	− 0.752***	−0.894***	−1.130***	−1.300***
	(0.048)	(0.057)	(0.061)	(0.077)	(0.090)	(0.114)
Hukou	−0.137	−0.154	−0.238	− 0.266	−0.082	0.256
	(0.181)	(0.201)	(0.215)	(0.242)	(0.329)	(0.407)
**Digital technology usage**						
Internet usage	−5.800**	−3.500*	−4.690***	−9.620***	−9.370**	−12.40***
	(2.890)	(2.070)	(1.680)	(2.910)	(4.140)	(3.890)
**Other**						
Age	0.014*	0.008	0.011	0.009	0.013	0.033*
	(0.007)	(0.008)	(0.009)	(0.011)	(0.013)	(0.017)
Gender	−1.330***	−1.290***	−1.360***	−1.970***	−2.190***	−2.790***
	(0.133)	(0.158)	(0.175)	(0.213)	(0.250)	(0.315)
Marital	−1.410***	−1.400***	− 1.610***	−1.970***	−2.050***	− 2.520***
	(0.161)	(0.212)	(0.235)	(0.307)	(0.290)	(0.414)
**Interaction effect**						
Internet usage * education	0.556	−1.460***	−0.142	0.765	4.570***	4.640*
	(2.400)	(0.546)	(0.719)	(1.230)	(1.450)	(2.620)
Internet usage * income	0.502**	0.472**	0.431**	0.816***	0.473	0.824*
	(0.206)	(0.211)	(0.179)	(0.271)	(0.439)	(0.449)
Internet usage * hukou	−0.760*	−0.550	−0.440	−0.369	−1.020	−2.680**
	(0.454)	(0.449)	(0.375)	(0.625)	(0.798)	(1.100)
Constant	16.500***	15.200***	18.000***	22.100***	27.200***	31.200***
	(0.753)	(0.900)	(0.951)	(1.210)	(1.400)	(1.760)
Observations	8853	8853	8853	8853	8853	8853
*R*^2^	0.110					
Pseudo *R*^2^		0.595	0.596	0.595	0.607	0.601

^a^ standardize coefficients are reported; standard errors in parentheses

^b^ ***p < 0.01, **p < 0.05, *p < 0.1

At high quantiles, the interaction effect of education and Internet usage is significantly positive (*β*_*DT* ∗ *EDU*_ = 4.570, *p* < 0.01, Q = 0.8 and *β*_*DT* ∗ *EDU*_ = 4.640, *p* < 0.1, Q = 0.9). Hence, Internet usage will negatively moderate the relationship between education and depression in the subgroup of severe depressive symptoms. The interaction effect of income and Internet usage remains significant, and the coefficient tends to increase, which indicates the strengthened negative moderation effect of Internet usage on the relationship between income and depression in the subgroup of severe depressive symptoms. In the scenario of Internet usage, **H4 is partly supported**.

The estimation of interaction model 2 see Additional file - Table 2, which evaluates the interaction effect of SES and mobile phone usage. The interaction effect of education and mobile phone usage is significantly negative on average condition (*β*_*DT* ∗ *EDU*_ =  − 0.561, *p* < 0.1, OLS). Hence, mobile phone usage will positively moderate the relationship between education and depression on average level. The direct effects of mobile phone usage at higher quantile levels (*β*_*DT*_) are not significant. And the interaction effects with income (*β*_*DT* ∗ *INCOME*_) and hukou (*β*_*DT* ∗ *HUKOU*_) also are not significant at all levels. In the scenario of mobile phone usage, **H3 and H4 are not supported**.

## Discussion

### Main findings

This study aims to (1) analyze the moderating role of digital technology usage on the relationship between SES and depression; and (2) explore the association of SES and depression as well as the moderating effect of digital technology in the subgroup of severe depressive symptoms. By using the China Health and Retirement Longitudinal Study 2015, our study yields three main findings. Our findings are summarized in Table 4.

**Table 4:** Summary of findings

Individual socioeconomic status	Disparity of depression			Digital technology intervention
	Average		Higher	
	Mean	Median	> 0.5 quantile	
Father’s education	NS^a^	NS	Positive	–^b^
Self-rated health status during childhood	Positive^c^	Positive	Increasing^d^	–
Education	Positive	Positive	Increasing	Strengthen (average)
				Weaken (higher)
Income	Positive	Positive	Increasing	Weaken
Hukou	Positive	Positive	Positive	NS

^a^*NS* not significant

^b^irreversible SES variables excluded from the interaction analysis

^c^The positive effect means that SES exacerbates health disparity, that is, higher SES leads to lower CES-D scores

^d^The increasing effect indicates the trend of SES effect on health disparity

First, H1 has partly supported means that at the average population level of later-life depressive symptoms, educated, higher income [5, 7, 8], Non-agricultural hukou (urban household registration) [9, 13], and better self-rated health status during childhood [8, 11] are associated with less likelihood of depression in middle-aged and aged individuals. At the average population level, the association between well parental education with less likelihood of depression might be mediated by the mid-life SES, such as education, income [11].

More importantly, H2 has partly supported. For the vulnerable subgroups with severe depressive symptoms, the protective effect of a good education, high income, and good self-rated health status during childhood on depressive symptoms will be enhanced. This effect of parental education and hukou also tends to increase. Thus, SES is correlated with the disparity in depression among middle-aged and aged individuals and reinforces this disparity under severe depression cases. Previous studies have verified disparity in depression of later life at the average population level [6–8, 30]. Using quantile regression, we focus on subgroups of severe depression symptoms and confirm that disparity in depression correlated with SES is larger for vulnerable groups.

Second, in the scenario of Internet usage, H3 and H4 have partly supported. The direct association between Internet usage and decline of depressive symptoms are in an agreement with prior studies [16, 20, 22, 23, 36, 39]. Based on the advantages of quantile regression, we further reveal this effect will be enhanced for the vulnerable subgroups with severe depressive symptoms.

On the average level, Internet usage will strengthen the association between a good education and less depressive symptoms, which means that Internet usage will increase the disparity of depression correlated with education. This finding might agree with the prior studies that well-educated individuals are more likely to access, use, and benefit from digital technology [45, 46]. More interestingly, for vulnerable subgroups with severe depressive symptoms, Internet usage will weaken the association between education and depressive symptoms, which means that SES disadvantaged subgroups also can relieve the depressive symptoms through Internet usage. At all quantile levels discussed in this study, Internet usage can weaken the association between high income and less depressive symptoms, which means that individuals suffering income-related disparity of depression can relieve the symptoms via digital technology. Thus, SES or health disadvantaged groups can obtain better mental health outcomes via digital technology usage [16, 20, 33, 36, 38, 47].

This result exhibits the advantage of quantile regressions over OLS [24, 25, 44]. At higher quantile levels of CES-D scores, we reveal the different relation of SES, depression, and digital technology usage. Previous studies have identified digital technology as a protective factor with the deterioration of depression status [20, 48]. Our results are consistent with this observation and provide additional insights on the role of digital technology. Besides the direct protective effect of digital technology on depression, it can also reduce the disparity in depression correlated with SES. This moderating effect becomes stronger for the vulnerable subgroups with severe depressive symptoms. Therefore, digital technologies are promising for controlling depression among older adults.

Finally, in the scenario of mobile phone usage, H3 and H4 have not been supported. Mobile phone ownership cannot reduce the disparity in depression correlated with SES. Although previous studies reported that mobile phone usage can improve health outcome [16, 18, 48], for the elderly in China, they may be more likely to hold regular mobile phones like other developing countries [49] and are unable to accept intelligent support via mobile phone.

### Practical implications

Our research has important implications for the practice of health disparity interventions and well-being of elderly.

Benefit from the advantages of quantile regression, we have expanded our sight to the subgroup of suffering more severe depressive symptoms among the Chinese middle-aged and older adults and confirmed that SES-related mental health disparity is more serious in that group. It is impossible for the policymakers to change the disadvantaged status of SES in the past, such as health status during childhood and parental education to alleviate the health disparity in later life. Instead, it is necessary to intervene in the current changeable factors actively.

First, the benefits of digital technology as a support system for reducing health disparity and well-being of older adults have also been confirmed in vulnerable subgroups suffering severe depressive symptoms. In Chinese middle-aged and older adults, Internet access is still limited to people with higher SES, such as well-educated, higher income, and urban residence; however, the mobile phone has been adopted by the general population [45]. Given the effectiveness of Internet use for well-being [16, 20, 22, 23, 33], the penetration rate of Internet in middle-aged and aged individuals needs to be improved. In addition to increasing the coverage of infrastructure in rural areas, it is even more necessary to increase Internet adoption among older adults in China. Providing Internet or digital technology training [35, 37, 50] and developing Gerontechnology [51, 52] can promote the integration of elderly into the digital world. Second, the value of mobile phone, which has a high penetration rate of digital technology to improve health outcome, is not utilized. The proven effectiveness of mobile health in developing health interventions [16, 18, 48] inspire us to launch large-scale delivery of health services through mobile phone. For example, providing social support for the elderly through low-cost short-message services for providers does not require Internet access and additional application to install for users [48, 53].

### Limitations and future research

Our research has the following limitations. First, the dataset used in this study is self-reported survey data, which might have measurement bias, especially items such as self-rated health status during childhood. Second, the survey includes two simple questions about digital technology usage, so we could measure only internet usage and mobile phone ownership. In this study, digital technology usage is a measurement of access to digital technology. In other words, the details of digital technology usage, such as frequency of use, purpose of use, and whether to use smartphone, are missing in our research. Future research can explore the mechanisms of digital technology usage impact on depression by obtaining detailed digital technology usage data.

## Conclusion

We explain how the individual socioeconomic status of middle-aged and aged individuals influence depression outcome and produce disparity and how digital technology moderates this disparity. The model is tested on cross-section data from the China Health and Retirement Longitudinal Study. We find evidence that individual socioeconomic status contributes to the emergence of later-life depression disparity, and digital technology moderates this connection. The result underscores the importance of social context of disparity in depression and the role of digital technology for improving the well-being of middle-aged and aged individuals.

## Supplementary Information

**Additional file 1.**

## Data Availability

Dataset from the China Health and Retirement Longitudinal Study (CHARLS) http://charls.pku.edu.cn/ .
