# Supplementary material for: Does digital technology reduce health disparity? Investigating difference of depression stemming from socioeconomic status among Chinese older adults
Source: BMC Geriatr. 2021 Apr 21;21:264. doi: 10.1186/s12877-021-02175-0 (PMC8059190; doi:10.1186/s12877-021-02175-0)
Supplement: Supplementary file 1 — Additional file 1. [file 12877_2021_2175_MOESM1_ESM.docx]

**Title:** Does digital technology reduce health disparity? Investigating difference of depression stemming from socioeconomic status among Chinese older adults

**Author Note:** Aruhan Mu, PhD student^1^, Zhaohua Deng, PhD^1^, Xiang Wu, PhD^1^, Liqin Zhou, PhD^1^*

^1^ School of Medicine and Health Management, Huazhong University of Science and Technology, Wuhan, China.

***Corresponding author:** Liqin Zhou*, School of Medicine and Health Management, Huazhong University of Science and Technology, Wuhan 430030, China. Email: [zhouliqin@hust.edu.cn](mailto:zhouliqin@hust.edu.cn). Tel: +8615927686325.

**Declarations:**

Ethics approval and consent to participate: Not applicable. Consent for publication: Not applicable. Competing interests: None.

Availability of data and materials: Dataset from the China Health and Retirement Longitudinal Study (CHARLS) <http://charls.pku.edu.cn/> .

Funding: This study was supported by the National Natural Science Foundation China (award no. 72004071).

Authors’ Contributions: Aruhan Mu (AM), Dr. Zhaohua Deng (ZD), Dr. Xiang Wu (XW), and Dr. Liqin Zhou (LZ) conceived the study. AM and XW organized the data and conducted the statistical analysis. AM drafted the first version of the manuscript, WX reviewed the data. ZD, XW, and LZ provided feedback on and contributed to subsequent versions of the manuscript. LZ provide suggestions and contributed to the first round of revision.

Acknowledgments: The authors are grateful to Dr. Ruoxi Wang at School of Medicine and Health Management, Huazhong University of Science & Technology, who gave valuable advices on this paper.

**Additional file:**

**Socioeconomic status and depression**

When we replacing variable “father’s education” with “mother’s education” to indicate parental education, model 1 is specified as

$$Doutcome=\beta_{0}+\beta_{1}{edu}_{mother}+\beta_{2}{srh}_{childhood}+\beta_{3}edu+ \beta_{4}income$$

$$+ \beta_{5}hukou +\beta_{6-8}control variables+e$$

where $\beta_{1},\beta_{2},\beta_{3},\beta_{4}, and \beta_{5}$ determine the effects of SES.

Table 1. *OLS analysis and quantile regression estimation for model 1*

| Variables | Dependent variable: depression (sample n = 8853) | | | | | |
| --- | --- | --- | --- | --- | --- | --- |
|  | OLS | Quantile regression  regression | | | | |
|  |  | 0.5 | 0.6 | 0.7 | 0.8 | 0.9 |
|  | (1) | (2) | (3) | (4) | (5) | (6) |
| **Individual socioeconomic status** | | |  |  |  |  |
| Mother's education | -0.224 | -0.234 | -0.268 | -0.371 | -0.406 | -0.481 |
|  | (0.130) | (0.173) | (0.202) | (0.253) | (0.320) | (0.355) |
| SHR-16 | -0.445*** | -0.457*** | -0.482*** | -0.565*** | -0.645*** | -0.681*** |
|  | (0.055) | (0.062) | (0.072) | (0.086) | (0.102) | (0.128) |
| Education | -0.845*** | -0.814*** | -1.010*** | -1.290*** | -1.860*** | -0.934** |
|  | (0.173) | (0.244) | (0.253) | (0.387) | (0.319) | (0.443) |
| Income | -0.666*** | -0.629*** | -0.761*** | -0.861*** | -1.100*** | -1.300*** |
|  | (0.047) | (0.056) | (0.060) | (0.076) | (0.090) | (0.100) |
| Hukou | -0.302* | -0.228 | -0.269 | -0.369* | -0.241 | -0.174 |
|  | (0.164) | (0.168) | (0.202) | (0.220) | (0.298) | (0.353) |
| **Other** | | | | |  |  |
| Age | 0.017** | 0.008 | 0.010 | 0.007 | 0.012 | 0.040** |
|  | (0.007) | (0.008) | (0.009) | (0.011) | (0.013) | (0.017) |
| Gender | -1.290*** | -1.230*** | -1.310*** | -1.950*** | -2.180*** | -2.650*** |
|  | (0.133) | (0.155) | (0.181) | (0.221) | (0.248) | (0.310) |
| Marital | -1.400*** | -1.340*** | -1.530*** | -1.860*** | -1.970*** | -2.620*** |
|  | (0.162) | (0.218) | (0.238) | (0.318) | (0.287) | (0.421) |
| Constant | 16.100*** | 15.100*** | 18.100*** | 22.100*** | 27.100*** | 31.000*** |
|  | (0.746) | (0.886) | (0.979) | (1.210) | (1.420) | (1.660) |
| Observations | 8,821 | 8,821 | 8,821 | 8,821 | 8,821 | 8,821 |
| R^2^ | 0.110 |  |  |  |  |  |
| Pseudo R^2^ |  | 0.597 | 0.598 | 0.596 | 0.610 | 0.604 |
| *Note.* ^a^ standardize coefficients are reported; standard errors in parentheses.  ^b^ ***p < 0.01, **p < 0.05, *p < 0.1. | | | | | | |

**Moderating effect of digital technology**

We use model 2 to investigate the moderating effect of digital technology usage including Internet usage and mobile phone usage, respectively. Table 2 shows the estimation of interaction effect of SES and mobile phone usage.

Table 2. *OLS analysis and quantile regression estimation for model 2 (mobile phone usage)*

| Variables | Dependent variable: depression (sample n = 8853) | | | | | |
| --- | --- | --- | --- | --- | --- | --- |
|  | OLS | Quantile regression  regression | | | | |
|  |  | 0.5 | 0.6 | 0.7 | 0.8 | 0.9 |
|  | (1) | (2) | (3) | (4) | (5) | (6) |
| **Individual socioeconomic status** | | | | |  |  |
| Father's education | -0.203 | -0.260* | -0.388** | -0.180 | -0.067 | -0.185 |
|  | (0.132) | (0.144) | (0.171) | (0.193) | (0.228) | (0.297) |
| SRH-16 | -0.442*** | -0.446*** | -0.510*** | -0.547*** | -0.650*** | -0.676*** |
|  | (0.055) | (0.062) | (0.074) | (0.085) | (0.098) | (0.121) |
| Education | -0.563** | -0.550* | -0.557 | -0.802 | -1.290*** | -0.846* |
|  | (0.228) | (0.308)^*^ | (0.355) | (0.499) | (0.446) | (0.507) |
| Income | -0.632*** | -0.620*** | -0.764*** | -0.871*** | -1.180*** | -1.310*** |
|  | (0.064) | (0.076) | (0.086) | (0.111) | (0.125) | (0.144) |
| Hukou | -0.377 | -0.254 | -0.206 | -0.387 | -0.408 | -0.109 |
|  | (0.239) | (0.227) | (0.279) | (0.319) | (0.400) | (0.523) |
| **Digital technology usage** | |  |  |  |  |  |
| Mobile phone usage | 1.210* | 1.260 | 1.140 | 0.764 | 0.250 | 0.088 |
|  | (0.671) | (0.868) | (0.944) | (1.260) | (1.290) | (1.590) |
| **Other** | | | | |  |  |
| Age | 0.019*** | 0.007 | 0.012 | 0.017 | 0.020 | 0.039** |
|  | (0.007) | (0.008) | (0.009) | (0.011) | (0.013) | (0.016) |
| Gender | -1.350*** | -1.320*** | -1.460*** | -1.980*** | -2.210*** | -2.660*** |
|  | (0.133) | (0.160) | (0.183) | (0.218) | (0.239) | (0.300) |
| Marital | -1.280*** | -1.300*** | -1.400*** | -1.820*** | -1.920*** | -2.610*** |
|  | (0.165) | (0.205) | (0.248) | (0.310) | (0.284) | (0.408) |
| **Interaction effect** |  |  |  |  |  |  |
| Mobile phone usage * education | -0.561* | -0.667 | -0.894* | -0.786 | -0.908 | -0.190 |
|  | (0.331) | (0.491) | (0.505) | (0.757) | (0.594) | (0.797) |
| Phone usage * income | -0.053 | -0.030 | 0.004 | 0.045 | 0.106 | 0.050 |
|  | (0.083) | (0.095) | (0.111) | (0.135) | (0.152) | (0.192) |
| Mobile phone usage * hukou | 0.026 | -0.136 | -0.242 | -0.230 | 0.214 | -0.404 |
|  | (0.318) | (0.312) | (0.379) | (0.415) | (0.518) | (0.703) |
| Constant | 15.400*** | 14.700*** | 17.600*** | 20.900*** | 26.500*** | 30.800*** |
|  | (0.839) | (0.992) | (1.140) | (1.420) | (1.560) | (1.940) |
| Observations | 8,853 | 8,853 | 8,853 | 8,853 | 8,853 | 8,853 |
| R^2^ | 0.110 |  |  |  |  |  |
| Pseudo R^2^ |  | 0.595 | 0.596 | 0.596 | 0.610 | 0.602 |
| *Note.* ^a^ standardize coefficients are reported; standard errors in parentheses.  Notes: ^b^ ***p < 0.01, **p < 0.05, *p < 0.1. | | | | | | |

Figure 1. *Effects of individual socioeconomic status on depression in older Chinese adults*


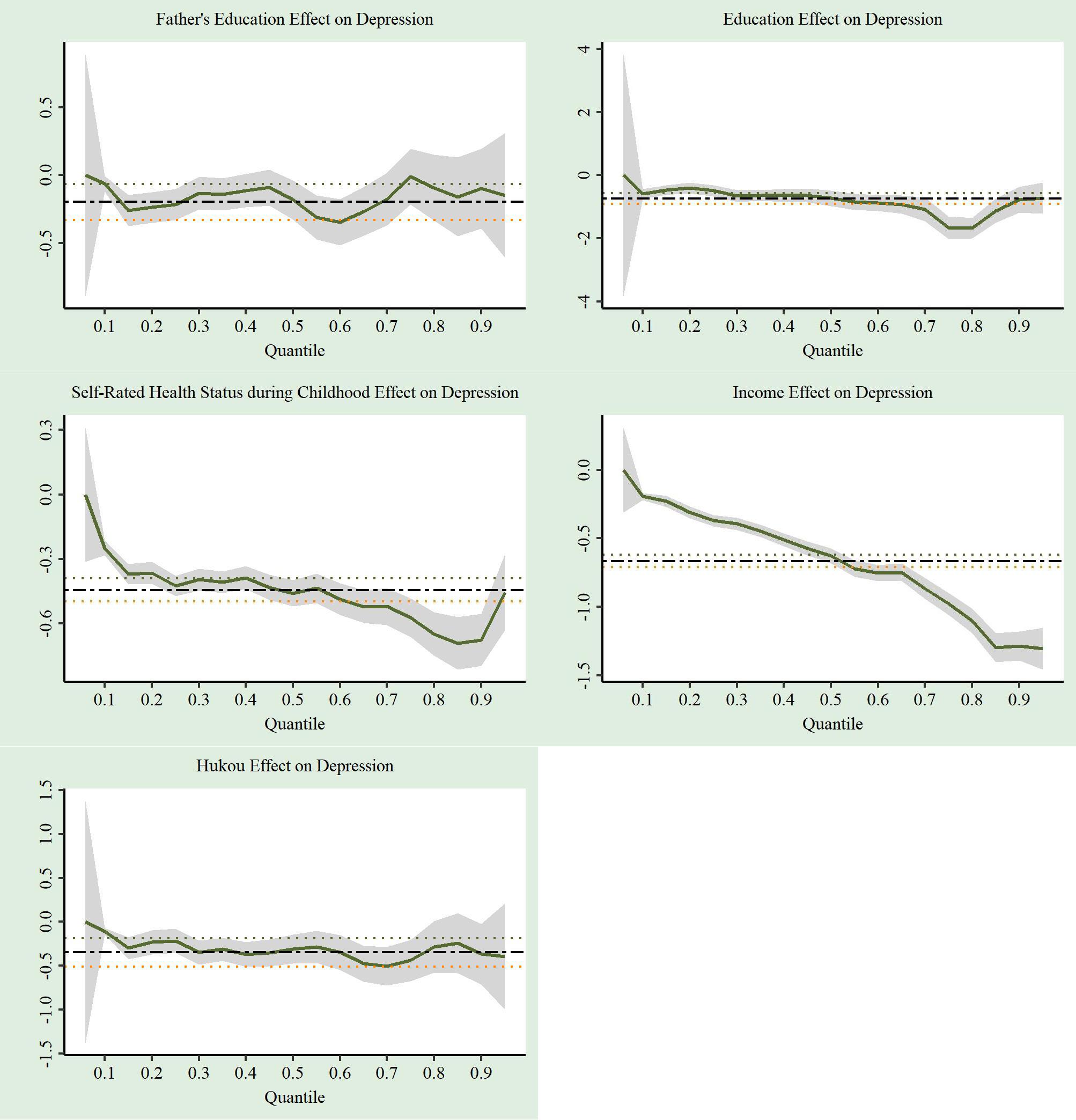


*Note.* The group shows the effects of individual socioeconomic status measures on depression CES-D score quantiles (green solid line). The x-axis is labeled with the quantile level at which the effects are estimated. The y-axis refers to the effect. The 95% confidence intervals of the effects on quantile are in the shaded area. The black dashed line refers the OLS effect of individual socioeconomic status at the mean CES-D scores.
